# Evaluation of Pain Through a Novel Mobile Application: The Visual, Auditory, and Tactile Scale (VATAS)

**DOI:** 10.7759/cureus.60793

**Published:** 2024-05-21

**Authors:** Ali Yüce

**Affiliations:** 1 Department of Orthopaedics and Traumatology, Prof. Dr. Cemil Taşcıoğlu Şehir Hastanesi, Istanbul, TUR

**Keywords:** mobile application, vatas, scale, tactile, visual, auditory, pain measurement, pain scale, pain rating, visual analog scale (vas)

## Abstract

Developing a validated, standardized, and easily accessible pain assessment tool is the first step toward improving pain management. Since pain measurement is often used as a primary or secondary endpoint in daily clinical practice and clinical trials, accurate and precise pain measurement is of great importance. Therefore, there is a need for a valid, reliable, safe, and low-cost method to measure and assess pain levels more objectively. Traditional measurement tools, still considered the gold standard in clinical pain research, have significant disadvantages, including low sensitivity and higher rates of false responses. Perhaps most importantly, the assumption that general pain is a one-dimensional experience that can be measured with a single-item scale is limiting. Recently, new technologies utilizing smart devices have emerged to improve existing traditional pain outcome measures. The Visual, Auditory, and Tactile Analog Scale (VATAS) was designed to address tactile, auditory, and visual senses. By including multiple senses, it is thought that errors arising from the objectification of pain solely through a single-dimensional scale, such as Visual Analog Scale (VAS), could be eliminated, providing a more standardized and repeatable pain assessment. VATAS has the potential to complement the deficiencies of standard pain measurement methods by appealing to multiple senses. It can provide a more standardized, patient-compliant, and repeatable pain assessment. Furthermore, it can be used for evaluating and recording pain in visually impaired patients and has the potential for data tracking, allowing patients' pain to be monitored even when they are at home.

## Introduction

Developing a validated, standardized, and easily accessible pain assessment tool is the first step towards improving pain management [1]. Since pain measurement is often used as a primary or secondary endpoint in daily clinical practice and clinical trials, accurate and precise pain measurement is of great importance [2]. The Visual Analog Scale (VAS), with its simple use, is one of the most commonly used pain assessment scales in daily clinical practice for pain measurement [2]. The increasing use of electronic medical records facilitates the transition of the VAS test from a paper-based format to a digital format, making it more suitable for monitoring and analyzing patient data [3]. Recently, new technologies utilizing smart devices have emerged to improve existing traditional pain outcome measures [4]. Therefore, in recent years, digital versions of the VAS have been evaluated in scientific studies, showing that they can increase usability and patient compliance [1, 3, 5]. Digital pain assessments, besides being portable and accessible, have the potential to reduce human errors that may occur during manual scoring and data recording [1, 4].

While the Visual Analog Scale (VAS) is widely used as a pain scale, it has recognized limitations when used as a data collection method based on the patient's self-report. It is considered to have good reliability and validity when the patient's self-report is used as the data collection method [6]. However, this simple pain scale has some limitations in assessing pain intensity. It is highly subjective and influenced by factors other than pain, such as mood [2]. Therefore, there is a need for a valid, reliable, safe, and low-cost method to measure and assess pain levels more objectively. Traditional measurement tools, still considered the gold standard in clinical pain research, have significant disadvantages, including low sensitivity and higher rates of false responses. Perhaps most importantly, the assumption that general pain is a one-dimensional experience that can be measured with a single-item scale is limiting [7]. Another limitation of the VAS is its conceptual complexity and the need to convert a sensory experience into a linear format. Some patients find the concept too abstract or difficult to understand, with reported rates of discordance ranging from 7% to 26% [6, 8]. From a clinical practice perspective, using reliable and valid methods to communicate the level of pain experienced by patients is an important part of quality patient care. However, using multiple pain measurement methods can be time-consuming and impractical in most acute situations. The evaluation of pain in clinical practice involves a general assessment of the individual's experience and represents a contextual judgment that guides clinical decision-making. This contrasts with the use of pain measurement methods in research, where the aim is often to measure the size and degree of pain experienced to allow for an unbiased comparison of results between patients or groups of patients [6].

Mobile versions of VAS and Numerical Rating Scale (NRS) are available [1]. In the literature, the usability of these mobile versions on different devices, the methods used to mark the location of pain, and the comparison of mobile versions with the classical method have been evaluated [1, 3-5, 7, 9]. Studies support the availability of mobile versions of these pain scales [1, 9]. VATAS is the first mobile pain scoring tool that addresses more than one sense.

## Technical report

Taking all of this into account, a pain scale that addresses both the auditory, visual, and tactile senses can benefit in increasing patient compliance and objectifying pain assessment, moving the single-dimensional experience measured by VAS to different dimensions (Video 1). 

**Video 1:** When the button is moved from 0 to 10 in VATAS: (1) The volume of the alarm sound increases; (2) The vibration intensity of the mobile device increases; (3) Screen brightness decreases; and (4) Screen background changes from blue to redScreen background changes from blue to red.

**Figure 1:**
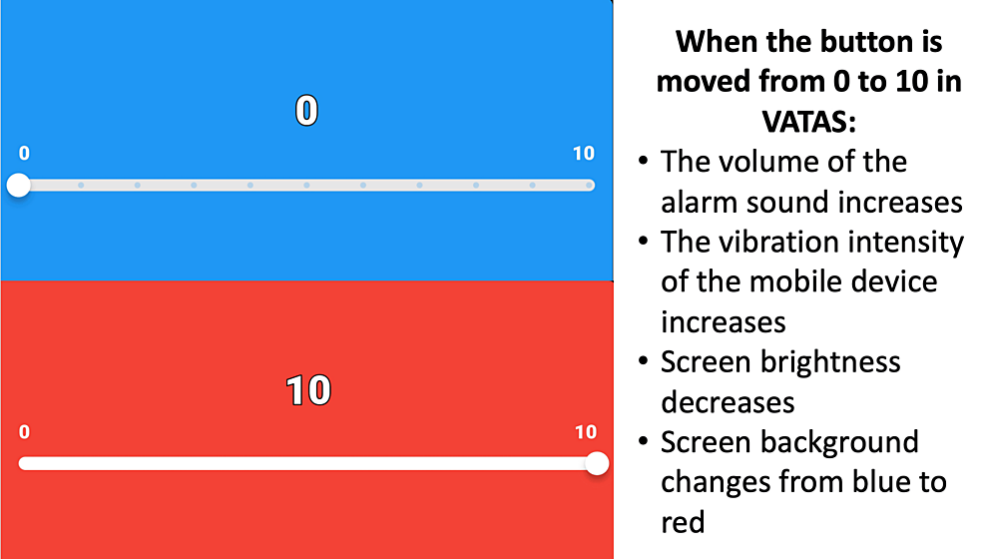
Features of VATAS. VATAS: Visual, Auditory, and Tactile Analog Scale.

The Visual, Auditory, and Tactile Analog Scale (VATAS) is a mobile application developed for pain assessment. Similar to the VAS, VATAS features a scale from zero to ten. The zero point on this scale represents no pain, while ten represents the highest level of pain. In VATAS, as the button on the scale is moved from zero to ten, an alert sound begins and its intensity increases gradually. Simultaneously, as the button moves from left to right on the scale, the vibration intensity of the mobile device increases. While the screen background of the mobile device is blue at zero, it changes color gradually, becoming red at ten. Additionally, when the button is at zero, the screen brightness of the mobile device is at its maximum, and as it moves towards ten, the screen brightness decreases (Figure 1). VATAS was designed to address tactile, auditory, and visual senses. By including these senses, it is thought that errors arising from the objectification of pain solely through a single-dimensional scale, such as VAS, could be eliminated, providing a more standardized and repeatable pain assessment. Moreover, instead of representing pain on a single line, the addition of color and screen brightness changes aimed to enhance the visual perception of pain representation. Finally, to increase ease of use, the feature of moving the button on the scale using the volume buttons on the mobile device was added, enabling marking on the scale both with the buttons and by moving the button on the screen.

Currently, VATAS is only compatible with Android-based mobile devices. For use in scientific studies, the application can be downloaded for free from the following link: 'www.vatas.com.tr'.

## Discussion

A digital-based pain scoring system, along with doctors, can provide improved patient communication. Data can be electronically recorded through web-based software that can be downloaded by patients and used in conjunction with an electronic medical record messaging system. It can facilitate the completion of patients' pain assessments and automatically send them to electronic medical records with a time and date stamp [3]. This way, healthcare providers can later access the results of each test and assess their patients' pain. This will not only allow real-time tracking of pain but also prevent potential recall bias. These data can provide more realistic pain expectations for patients scheduled to undergo the same procedure [3]. As of now, the VATAS does not have the feature of data tracking and recording. However, after its reliability, validity, and repeatability are proven through scientific studies, it can evolve into a form that can be used for recording and tracking pain in patients.

VAS and NRS have high correlation and are probably the most commonly used pain scales for measuring pain in daily clinical practice [2, 8, 10]. However, these simple pain scales have some limitations in assessing pain intensity. They are highly subjective and dependent on many factors other than pain, such as mood [2]. VATAS, although it uses a visual ruler as its basic tool and is named after VAS, is an 11-point scale ranging from 0 to 10. This feature is similar to the 11-point NRS. The mobile device versions of VAS and NRS have been proven to be as successful as their originals [1]. Chiu LY et al. have noted that there may be exciting results for remote pain assessment and management applications, including smartphone versions of VAS and NRS [1]. VATAS is designed to be a mobile scale for the improved assessment of pain. The usability and success of VATAS must be proven through scientific studies. Scientific studies comparing VATAS to standard pain scales are needed. Once the validity of VATAS is scientifically proven, data recording features such as recording the times of painkiller use and recording the date and time when pain is assessed can be added to VATAS. In this way, more data on pain can be obtained, such as the frequency, severity, and quality of the patient's pain and its relationship with treatments. Tracking and analyzing this data can help us better understand patients' pain. As a result, patient-specific treatment approaches can be developed.

In a study, the primary reason for patients not completing the VAS score was shown to be that patients did not understand the VAS and could not use it due to visual impairments, accounting for 73% of cases [6]. Evaluating pain perception in children is generally challenging. Additionally, appropriate treatment of pain requires the proper assessment of pain severity and the ability of children to describe the intensity of pain accurately. This issue has been addressed through the development of age-appropriate pain scales [11]. VATAS may increase compliance in the assessment of pain in elderly patients (especially those unable to participate in the assessment due to visual impairments). Scientific studies are also needed to evaluate the success of VATAS in children. Based on the results of these scientific studies, VATAS can be modified to increase compliance and be made more engaging for children by modifying visual and auditory stimuli.

Due to its one-dimensional nature, VAS is successful in acute pain but inadequate in representing pain in chronic pain conditions [12]. VATAS could be an alternative to solve this problem. Additionally, VATAS could carry the advantages of data tracking, evaluation without records, and reducing human errors. Blind people perceive the world by touching with their fingers, and it is important to understand their tactile perception [13, 14]. Therefore, visual deprivation also affects pain perception [14]. The study of blindness shows that pain and other nociceptive responses emerge from nervous processing mechanisms that integrate visual information. Moreover, the experiments reviewed here show that the protective function of nociception depends on its ability to interact with vision (and probably hearing and touch) [13]. To our knowledge, there is no pain assessment system specifically for visually impaired people. VATAS can be used as a purely auditory and tactile scale in visually impaired patients. Considering this, features have been added to the scale that can guide the user through the scale using the phone's keys. Finally, while focusing on pain in a one-dimensional way is limiting, using multiple scales is not feasible in terms of applicability. VATAS has the potential to be an alternative pain measurement method to address this issue in pain assessment. VATAS is a newly designed application and no scientific study has yet been conducted to confirm its applicability. Despite being an improvement over VAS, VATAS may have limitations in the clinical setting. VAS is a simple tool and can easily be accessed either on the patient information whiteboard in the hospital room, or in the clinic, whereas with VATAS being software, every time a healthcare practitioner (nurse, physical therapist, doctor) wants to assess pain, they have to open an application on their phone or tablet computer, which may be difficult if the patient is in isolation. Therefore, studies evaluating the validity of VATAS and its applicability in clinical settings are needed.

The features of VATAS can be developed after its applicability in studies with patients is proven. For example, the characteristic features of pain (colicky pain, dull, sharp, neuropathic, etc.) can be simulated with sound and/or vibration features. After determining the sound and vibration preferences representing the patient's pain, the patient can indicate their pain on the scale using the selected sound and vibration. This will also allow us to understand the patient's pain more. Additionally, after the usability of VATAS in pediatric patients is proven, the background images, sound, and vibration of VATAS can be designed to be suitable and engaging for children.

## Conclusions

VATAS has the potential to complement the deficiencies arising from the one-dimensional nature of standard pain measurement methods by appealing to multiple senses. It can provide a more standardized, patient-compliant, and repeatable pain assessment. Furthermore, it can be used for evaluating and recording pain in visually impaired patients. It has the potential for data tracking, allowing patients' pain to be monitored even when they are at home. However, scientific evidence is needed to demonstrate this. Further studies are needed to prove the repeatability, patient compliance, usability in chronic patients, usability in children and the elderly, and whether this scoring system is superior to standard pain scoring systems. According to the results of these studies, VATAS has the potential to be redesigned and developed.
